# Investigation of an FGFR-Signaling-Related Prognostic Model and Immune Landscape in Head and Neck Squamous Cell Carcinoma

**DOI:** 10.3389/fcell.2021.801715

**Published:** 2022-02-14

**Authors:** Qi Chen, Ling Chu, Xinyu Li, Hao Li, Ying Zhang, Qingtai Cao, Quan Zhuang

**Affiliations:** ^1^ Transplantation Center, Third Xiangya Hospital, Central South University, Changsha, China; ^2^ Xiangya School of Medicine, Central South University, Changsha, China; ^3^ Xiangya School of Stomatology, Central South University, Changsha, China; ^4^ Department of Pathology, Third Xiangya Hospital, Central South University, Changsha, China; ^5^ Hunan Normal University School of Medicine, Changsha, China; ^6^ Research Center of National Health Ministry on Transplantation Medicine, Changsha, China

**Keywords:** head and neck squamous cell carcinoma, fibroblast growth factor receptor, hypoxia, glycolysis, prognosis, immune-cell infiltration

## Abstract

**Background:** There is accumulating evidence on the clinical importance of the fibroblast growth factor receptor (FGFR) signal, hypoxia, and glycolysis in the immune microenvironment of head and neck squamous cell carcinoma (HNSCC), yet reliable prognostic signatures based on the combination of the fibrosis signal, hypoxia, and glycolysis have not been systematically investigated. Herein, we are committed to establish a fibrosis–hypoxia–glycolysis–related prediction model for the prognosis and related immune infiltration of HNSCC.

**Methods:** Fibrotic signal status was estimated with microarray data of a discovery cohort from the TCGA database using the UMAP algorithm. Hypoxia, glycolysis, and immune-cell infiltration scores were imputed using the ssGSEA algorithm. Cox regression with the LASSO method was applied to define prognostic genes and develop a fibrosis–hypoxia–glycolysis–related gene signature. Immunohistochemistry (IHC) was conducted to identify the expression of specific genes in the prognostic model. Protein expression of several signature genes was evaluated in HPA. An independent cohort from the GEO database was used for external validation. Another scRNA-seq data set was used to clarify the related immune infiltration of HNSCC.

**Results:** Six genes, including AREG, THBS1, SEMA3C, ANO1, IGHG2, and EPHX3, were identified to construct a prognostic model for risk stratification, which was mostly validated in the independent cohort. Multivariate analysis revealed that risk score calculated by our prognostic model was identified as an independent adverse prognostic factor (*p* < .001). Activated B cells, immature B cells, activated CD4^+^ T cells, activated CD8^+^ T cells, effector memory CD8^+^ T cells, MDSCs, and mast cells were identified as key immune cells between high- and low-risk groups. IHC results showed that the expression of SEMA3C, IGHG2 were slightly higher in HNSCC tissue than normal head and neck squamous cell tissue. THBS1, ANO1, and EPHX3 were verified by IHC in HPA. By using single-cell analysis, FGFR-related genes and highly expressed DEGs in low-survival patients were more active in monocytes than in other immune cells.

**Conclusion:** A fibrosis–hypoxia–glycolysis–related prediction model provides risk estimation for better prognoses to patients diagnosed with HNSCC.

## Introduction

Head and neck squamous cell carcinoma (HNSCC) has a worldwide incidence of more than 600,000 cases per year (Ferlay et al., 2015), including a heterogeneous group of tumors that arise from the oral cavity, oropharynx, larynx, hypopharynx, nasopharynx, and sinonasal cavity (Kim et al., 2021). Due to its special anatomical location, patients with HNSCC often experience vital dysfunction, especially in the aspects of swallowing, feeding, breathing, and psychological health (Zhu et al., 2017). Despite significant progress in available therapies, the 5-year overall survival (OS) rate of HNSCC patients has not obviously improved in recent decades (Siegel et al., 2019). Therefore, there is an urgent need for a way to predict the progression of HNSCC (Shield et al., 2017).

The fibroblast growth factor receptor (FGFR) is a receptor tyrosine kinase (RTK) signaling pathway involved in the regulation of angiogenesis, invasion, and metastasis of tumors (Chae et al., 2017). It is considered to be a contributory factor in the development of fibrosis, causing the exacerbation of liver fibrosis (Seitz and Hellerbrand, 2021), systemic sclerosis (SS) (Chakraborty et al., 2020), idiopathic pulmonary fibrosis (IPF) (Wollin et al., 2015), and so on. A recent study also revealed the clinically activity of an FGFR inhibitor against HNSCC (Schuler et al., 2019). However, few investigations focus on the potential mechanisms about this profibrotic mediator in HNSCC.

Hypoxia, as a hallmark of tumor, is a potent microenvironmental factor facilitating proliferation and progression in a variety of cancers (Rankin and Giaccia, 2016). Previous research suggests that hypoxic HNSCC cells trigger glycolysis to obtain energy and balance metabolic and bioenergetic (Zhu et al., 2017). Furthermore, activated fibroblasts synthesized excessive collagen, leading to a microenvironment of hypoxia, which might deteriorate the progression of disease, whereas there were few records on the prognosis of HNSCC by combining a profibrotic signal with hypoxia and glycolysis. The immune landscape associated with the factors mentioned above also demands exploration.

Therefore, in this study, we establish an FGFR-signaling–hypoxia–glycolysis–related prediction model for the prognosis of HNSCC with a series of bioinformatics analyses. Immune cell infiltration associated with prognosis and profibrotic signaling is also revealed.

## Methods

### Patient Cohort and Data Preparation

The discovery cohort of the study contained 483 HNSCC patients from the Cancer Genome Atlas (TCGA, available at https://portal.gdc.cancer.gov/) data set. To obtain a validation cohort, RNA-seq data and related clinicopathological data were downloaded from the Gene Expression Omnibus (GEO, available at https://www.ncbi.nlm.nih.gov/geo/) database (GSE41613), including 97 patients with HPV-negative oral squamous cell carcinoma (OSCC). The microarray data of GSE41613 was built upon the GPL570 Platform (Affymetrix Human Genome U133 Plus 2.0 Array). A single-cell RNA sequencing (scRNA-seq) data set (GSE139324) was also used for analyzing tumor immune infiltration in HNSCC patients, including tumor-infiltrating immune cells from 18 HNSCC patients and tissue resident immune cells from five healthy donor tonsils (Cillo et al., 2020).

**FIGURE 1:**
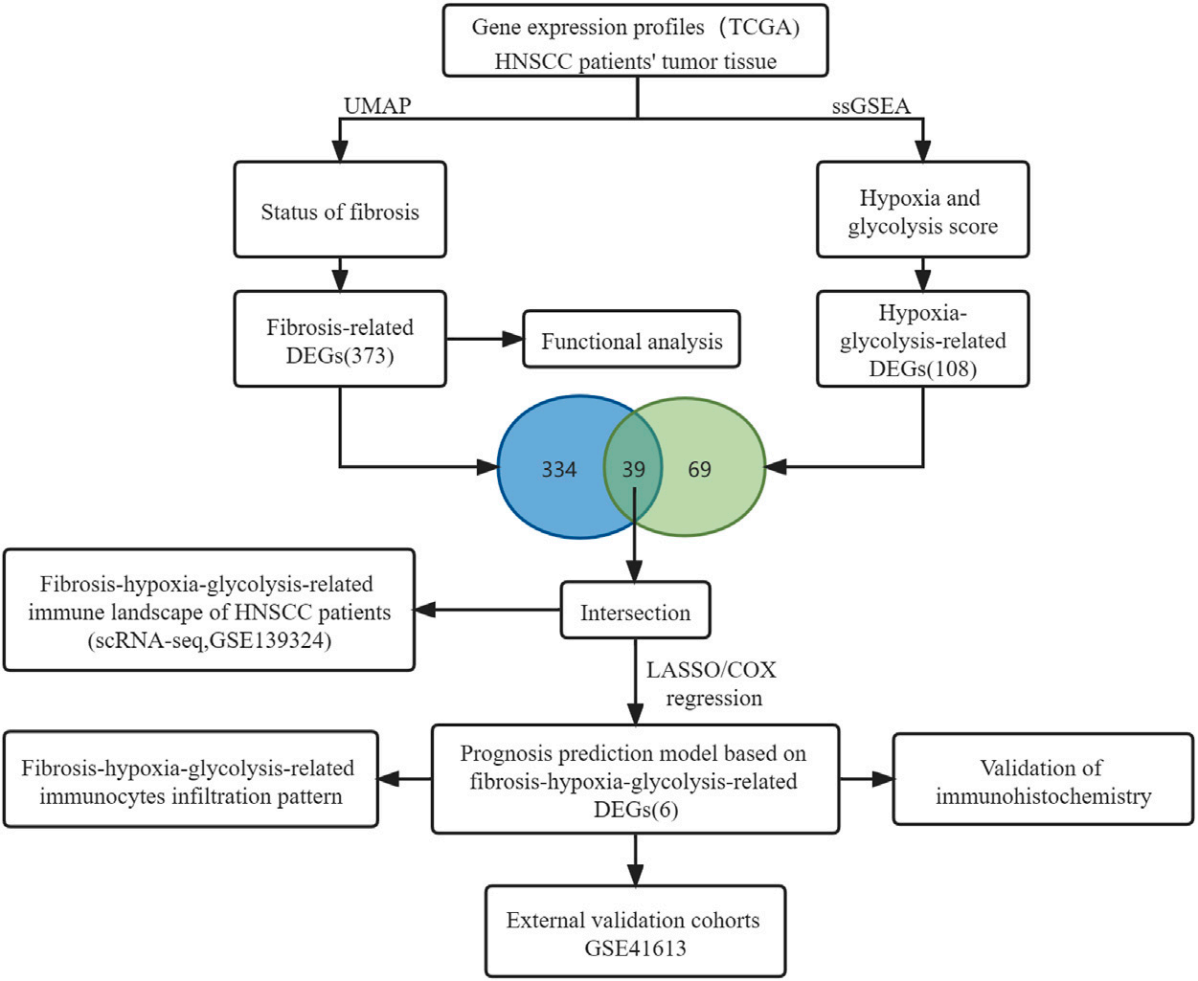
Flowchart diagram of the study.

The mRNA expression profiles from the TCGA database were normalized using fragments per kilobase of exon per million reads mapped (FPKM). Background correction and normalization has been performed for each series before downloading GSE41613 from the GEO database. Additionally, the harmony algorithm was used in the integration of the single-cell RNA-seq data set GSE139324 considering biological and technical differences (Korsunsky et al., 2019). The general idea and methodologies of our study are shown in a flowchart (Figure 1).

### Distinction of Fibrotic Signal Status and FGFR-Related DEGs

An algorithm of uniform manifold approximation and projection (UMAP) was applied to deduce the fibrotic signal status of HNSCC patients. Based on the given hallmarks or signatures, UMAP, a nonlinear reductive dimension method, is able to assign a group of patients to diverse clusters. The gene set of the FGFR signal pathway was downloaded from the Molecular Signatures Database (MSigDB version 6.0), identifying the relative activation degree of fibrotic signal in patients. Based on the limma algorithm (Korsunsky et al., 2019) and functional enrichment analysis, two clusters including “fibrosis^low^” and “fibrosis^high^” were identified to estimate the fibrotic signal status. The limma algorithm was applied to identify DEGs between the two clusters based on the standards of false discovery rate (FDR) adjusted *p*-value <.0001 and | log2(Fold change) | > 1. To confirm biological functions and pathway enrichment of the fibrosis-related DEGs, we performed Gene Ontology (GO) functional analysis and Kyoto Encyclopedia of Genes and Genomes (KEGG) pathway enrichment analysis using the “Metascape” website (https://metascape.org/) (Zhou et al., 2019).

### Distinction of Hypoxia–Glycolysis–Related DEGs

The ssGSEA algorithm was applied to explore the hypoxia and glycolysis degree in the HNSCC expression profile of the TCGA database. According to hypoxia and glycolysis scores calculated previously, two groups of patients were stratified. An optimal cutting point for classifying was determined by maximally selected rank statistics using the “survival” and “survminer” R package. Subsequently, “hypoxia^high^,” “hypoxia^low^,” “glycolysis^high^,” and “glycolysis^low^” groups were identified, respectively. We further considered hypoxia and glycolysis together by combining them into a two-dimensional index; that is, patients were divided into three groups, i.e., hypoxia^low^/glycolysis^low^, hypoxia^high^/glycolysis^low^, and “mix” groups. Hypoxia–glycolysis–related DEGs were identified based on the standards of FDR adjusted *p*-value <.0001 and | log2(Fold change) | > 1 using the R package “limma”.

### Prognosis Prediction Model of HNSCC Based on Fibrosis–Hypoxia–Glycolysis–Related DEGs

Hypoxia–glycolysis–related DEGs and fibrosis–related DEGs were intersected to obtain the 39 shared fibrosis–hypoxia–glycolysis–related DEGs by Venn analysis. To obtain prognostic shared DEGs, univariate Cox regression analyses were further performed among all 39 DEGs mentioned above to screen those risk or protective shared DEGs with *p* < .05. Thereafter, we applied the least absolute shrinkage and selection operator (LASSO) (Friedman et al., 2010; Liu et al., 2013) to preserve valuable variables in 21 prognostic shared DEGs, implementing a high-dimensional prediction and avoiding overfitting. The LASSO Cox regression model was scientifically built up depending on threefold cross-validation and 1000 iterations, which decreased the underlying instability of the results. The optimal tuning parameter λ was identified *via* 1-SE (standard error) criterion. Eventually, the selected prognostic gene signatures were used to establish the prognosis prediction model of HNSCC based on fibrosis–hypoxia–glycolysis–related DEGs. The risk score computing formula is:

$$Risk\;score=\overset{n}{\underset{i=1}{\sum }}\left(coefficient_{i}\times \exp ression\;of\;signature\;gene_{i}\right).$$

Based on the risk scores, we computed the optimal cutting point to stratify HNSCC patients into high- and low-risk groups.

### Identification of Immune Cell Infiltration Status

To predict the immune cell infiltration status, the ssGSEA algorithm was applied to identify the abundance of 28 immunocytes in each HNSCC patient, confirming the underlying correlation between immune infiltration status and prognostic model. The gene set from Charoentong et al. was obtained to calculate ssGSEA scores for immune cell populations. (Charoentong et al., 2017).

### Evaluation of Immunohistochemical Staining

Formalin-fixed, paraffin-embedded tumor tissues were collected from eight patients with HNSCC diagnosed at the 3rd Xiangya Hospital from January 2020 to December 2020. This study was approved by the Ethics Committee of the 3rd Xiangya Hospital (No: 21158). The immunohistochemical process was performed as described previously (Guan et al., 2020). Our procedure used the following antibodies: Polyclonal rabbit anti-Semaphorin 3c (1:200 dilution; ab135842; Abcam Biochemicals, UK); monoclonal rabbit anti-human IgG2 (1:1000 dilution; ab134050; Abcam Biochemicals, UK). IHC results for IGHG2 and SEMA3C were evaluated by computerizing optical density (OD) measurements using ImageJ software, which depends on the degree and area of staining. Samples were scored by two trained pathologists according to the percentage contribution of high positive, positive, low positive, and negative. The immunoreactive score (IRS) was evaluated as follows: 4, high positive; 3, positive; 2, low positive; and 1 negative (Varghese et al., 2014).

The Human Protein Atlas (HPA, https://www.ptroteinatlas.org/) provides us the IHC staining data in HNSCC and normal tissue (Uhlen et al., 2010). The expression level of target protein was classified into high, medium, low, and not detected according to degree of staining (strong, moderate, weak, or negative) and the proportion of stained cells (>75%, 25%–75%, or <25%).

### Single-Cell Analysis of Tumor Infiltrating Immune Cells From HNSCC Patients

After dimension reduction through principal component analysis (PCA), the *t*-distributed stochastic neighbor embedding (t-SNE) algorithm (Kobak and Berens, 2019), a technique that maps a set of high-dimensional points to two dimensions, was used to compute the degree of similarity between cells, which is visualized by the distances among the plotted points on the graph. It also potently governed how many of its nearest neighbors each point is attracted to. Here, each point represented a cell. The scores of individual cells for pathway activities were estimated by the R package “AUCell.” According to gene expression rankings in each cell for a certain gene set, area under the curve (AUC) values were calculated to represent the proportion of top-ranking genes in the gene set for each cell (Corridoni et al., 2020).

### Statistical Analysis

Using R version 4.0.2 (www.r-project.org/) and the appropriate packages, all statistical analysis was carried out. We implemented the UMAP algorithm using R package “umapr” for nonlinear dimension reduction and the ssGSEA algorithm using R package “GSVA” for the hypoxia and glycolysis score. The Lasso Cox regression model was conducted, and standard statistical tests were guaranteed by using the R package “glmnet.” The FDR method was performed to adjust multiple tests. Risk factors were eventually identified through multivariate Cox regression analysis.

## Results

### Fibrosis Signal and Fibrosis-Related DEGs in HNSCC

**FIGURE 2:**
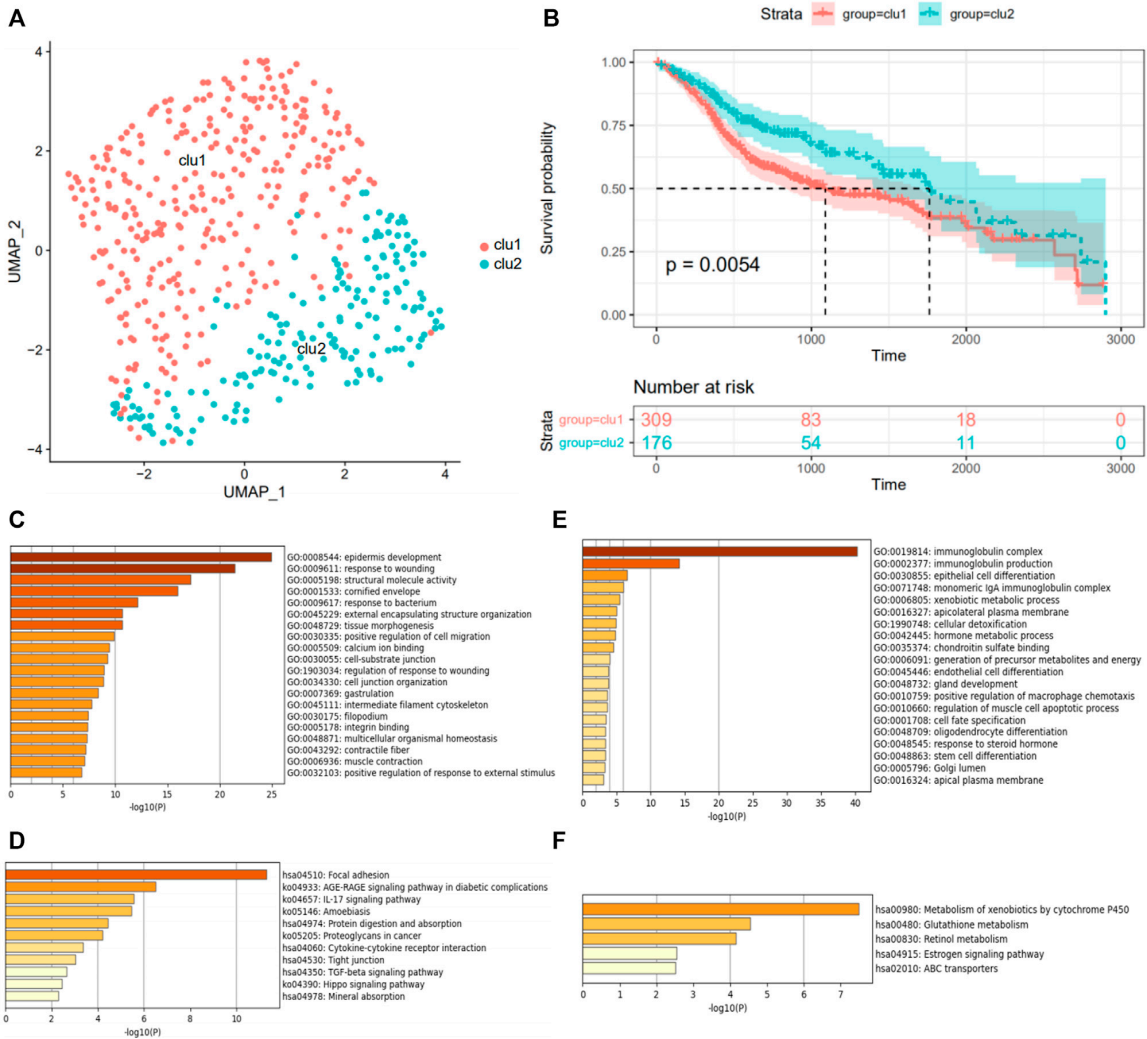
Grouping of patients according to FGFR signaling pathway. **(A)** The UMAP algorithm classifies HNSCC patients into two clusters, indicated by different colors. **(B)** The survivorship analysis plot of OS in two clusters. GO function enrichment **(C,E)** and KEGG pathway enrichment analysis **(D,F)** of differentially expressed genes in the fibrosis^**low-survival**^ group, colored by *p*-values. **(C,D)** Analysis of the high expression genes in the fibrosis^**low-survival**^ group. **(E,F)** Analysis of the low expression genes in the fibrosis^**low-survival**^ group.

The expression profiles and clinical information of 483 HNSCC patients were contained in the discovery cohort downloaded from the TCGA database. The clinical information of patients is shown in Table 1. Seventy genes positively correlated with the FGFR signaling pathway were used to evaluate the status of fibrosis signal activation in patients. Based on the algorithm UMAP, we divided the patients into two clusters using the fibrosis-related expression matrix, enabling us to assign each patient to the nearest cluster (Figure 2A). A Kaplan–Meier plot demonstrates that significant differences in survival were witnessed between the two clusters (Figure 2B). There were 309 and 176 patients included in clusters 1 and 2, respectively. To obtain fibrosis-related DEGs, we compared the expression profiles between the clusters. A total of 187 fibrosis-related DEGs overexpressed in cluster 1, where patients had worse survival, which were enriched in “response to wounding” (Figure 2C), “TGF-beta signaling pathway” (Figure 2D). This implied the patients in cluster 1 were in a higher state of fibrosis activation. Enrichment analysis showed 186 DEGs overexpressed in cluster 2 were enriched in “immunoglobulin complex” (Figure 2E), “metabolism of xenobiotics cytochrome P450” (Figure 2F). These findings are consistent with the previous research that patients with good immune status have a better prognosis.

**TABLE 1:** Basic information of HNSCC patients in discovery cohort.

Characteristics	Whole cohort (483)	Low risk (327)	High risk (156)
Gender			
Male	355 (0.735)	246 (0.752)	109 (0.699)
Female	128 (0.265)	81 (0.248)	47 (0.301)
Age			
≥60 years	156 (0.323)	190 (0.581)	80 (0.513)
<60 years	213 (0.441)	137 (0.419)	76 (0.487)
original diagnosis			
Squamous cell carcinoma, NOS	409 (0.847)	272 (0.832)	137 (0.878)
Squamous cell carcinoma, keratinizing, NOS	52 (0.108)	33 (0.101)	19 (0.122)
Squamous cell carcinoma, large cell, nonkeratinizing, NOS	11 (0.023)	11 (0.034)	0 (0.000)
Basaloid squamous cell carcinoma	10 (0.021)	10 (0.031)	0 (0.000)
Squamous cell carcinoma, spindle cell	1 (0.002)	1 (0.003)	0 (0.000)
UMAP clustering			
Cluster1	309 (0.640)	173 (0.529)	136 (0.872)
Cluster2	174 (0.360)	154 (0.471)	20 (0.128)
Hypoxia status			
High	381 (0.789)	242 (0.740)	139 (0.891)
Low	102 (0.211)	85 (0.260)	17 (0.109)
Glycolysis status			
High	318 (0.658)	189 (0.578)	129 (0.827)
Low	165 (0.342)	138 (0.422)	27 (0.173)
Risk group			
High	156 (0.323)	0 (0.000)	156 (1.000)
Low	327 (0.677)	327 (1.000)	0 (0.000)

### Hypoxia Status, Glycolysis Status, and Hypoxia–Glycolysis–Related DEGs in HNSCC

**FIGURE 3:**
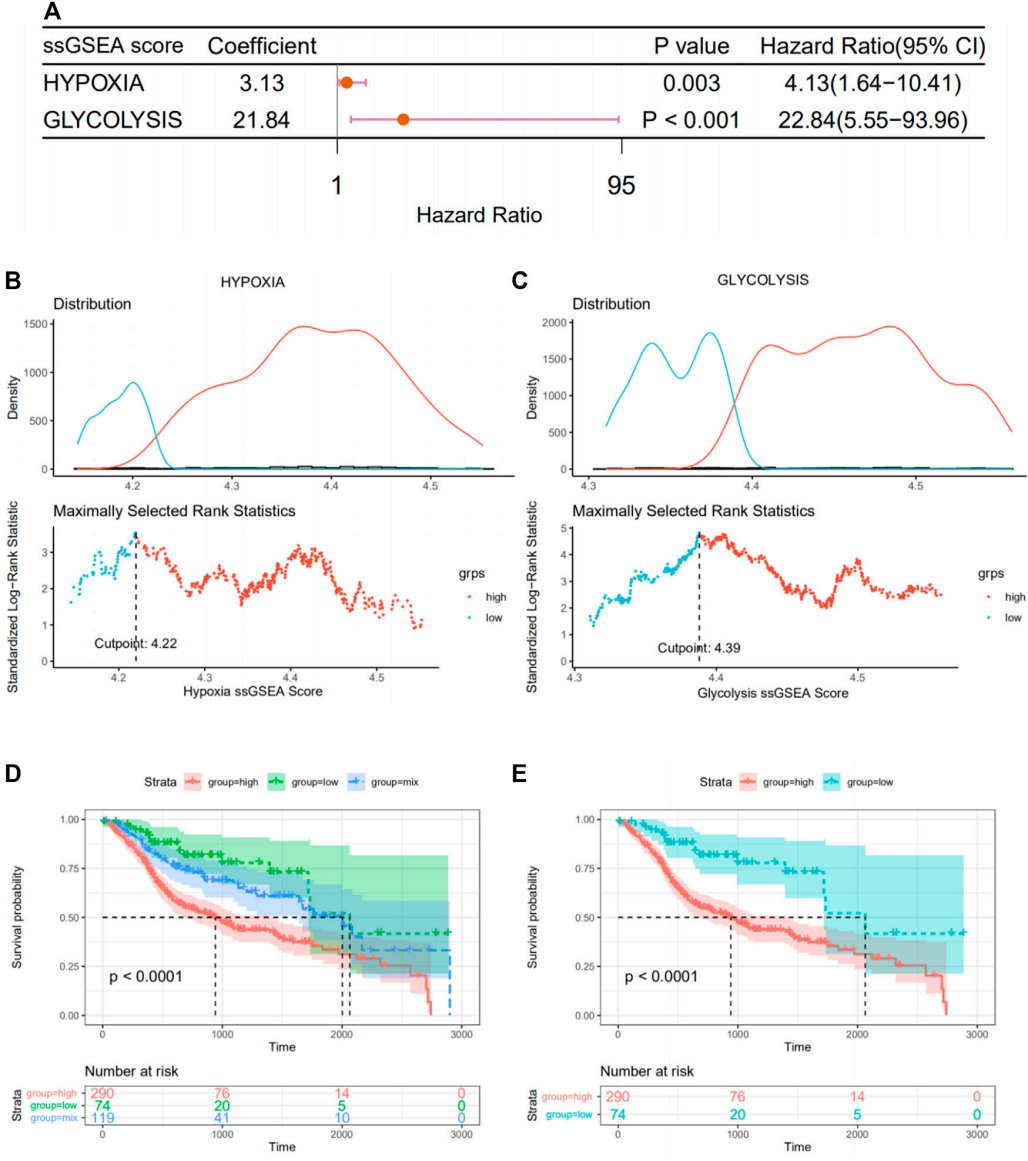
Grouping of patients according to their hypoxia and glycolysis status. **(A)** Forest plot of hypoxia and glycolysis scores by univariate Cox regression. **(B,C)** A vertical dashed line was used to mark the cutoff point with the maximum standard log-rank statistic based on hypoxia and glycolysis scores. **(D)** Kaplan–Meier plot of OS among hypoxia^**high**^/glycolysis^**high**^, hypoxia^**low**^/glycolysis^**low**^, and mix groups. **(E)** Kaplan–Meier plot of OS between hypoxia^**high**^/glycolysis^**high**^ and hypoxia^**low**^/glycolysis^**low**^.

Meanwhile, using the “GSVA” package, the ssGSEA algorithm was implemented to quantify the hypoxia or glycolysis enrichment score of each HNSCC patient in hypoxia or glycolysis hallmark genes from the MSigDB. To identify the effect of hypoxia and glycolysis on prognosis, univariate Cox regression analyses were further performed among patients’ hypoxia and glycolysis scores. Hypoxia and glycolysis, as illustrated in the forest diagram in Figure 3A, were considered risk factors for prognosis in HNSCC patients. Based on maximally selected rank statistics, we divided patients into two groups according to hypoxia (Figure 3B) and glycolysis (Figure 3C) scores. We further synthesized the hypoxia and glycolysis status into a two-dimensional index, dividing patients into three groups, i.e., hypoxia^high^/glycolysis^high^, hypoxia^low^/glycolysis^low^, and “mix” groups. Significant differences in survival were observed among three groups (Figure 3D, log rank test, *p* < .0001). The survivorship analysis (Kaplan–Meier) showed a better survival in the hypoxia^high^/glycolysis^high^ group than the hypoxia^low^/glycolysis^low^ group as we expected (Figure 3E). Besides this, the mix group was at an intermediate level. A total of 108 hypoxia–glycolysis–related DEGs were obtained after comparing expression profiles between hypoxia^high^/glycolysis^high^ and hypoxia^low^/glycolysis^low^.

### Construction of the Fibrosis–Hypoxia–Glycolysis–Related Prognostic Model in the TCGA Data set

**FIGURE 4:**
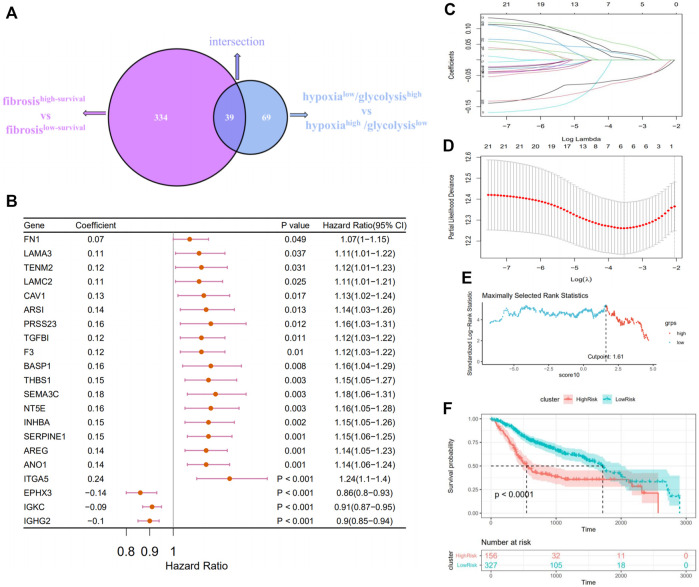
Constructing a prognostic survival model in patients with HNSCC. **(A)** Venn diagrams show the fibrosis–hypoxia–glycolysis related DEGs (39). **(B)** Forest plot of the prognostic effects of 21 DEGs with *p* < .05 on a univariate Cox regression analysis. **(C)** LASSO coefficient profiles of 21 screened DEGs. **(D)** Threefold cross-validation for LASSO analysis was performed to calculate the minimum criteria. **(E)** A vertical dashed line was used to mark the cutoff point with the maximum standard log-rank statistic. **(F)** Kaplan–Meier plot established the survival differences between high- and low-risk groups.

The above 334 fibrosis-related and 108 hypoxia–glycolysis–related DEGs screened from HNSCC were intersected to obtain the 39 shared genes (Figure 4A). To further filtrate the prognostic DEGs, univariate Cox regression analysis was conducted on 39 shared DEGs, and 21 DEGs with *p* < .05 were identified (Figure 4B). Among them, most of them (18 out of 21, 85.7%) were risk DEGs. Six critical variables were selected from the above 21 prognostic DEGs using the LASSO regression method, among which four were risk DEGs and two were protective (Figures 4C,D). For each HNSCC patient, a risk score was calculated based on the expression levels of the six characteristic DEGs and corresponding coefficients from the LASSO Cox regression: risk score = 0.0369 × expression of AREG+ 0.03432 × expression of THBS1+ 0.02182 × expression of SEMA3C+ 0.07125 × expression of ANO1+(-0.07718) × expression of IGHG2+ (-0.09177) × expression of EPHX3. Using the maximum selective rank method as the basis of demarcation, patients were divided into high- and low-risk groups according to their risk scores (Figure 4E). The low-risk group showed a significantly better effect on prognosis compared with the high-risk group (Figure 4F, log rank test, *p* < .0001).

### Supplementary Information on Prognostic Model and its Relationship With Immunocyte Infiltration

**FIGURE 5:**
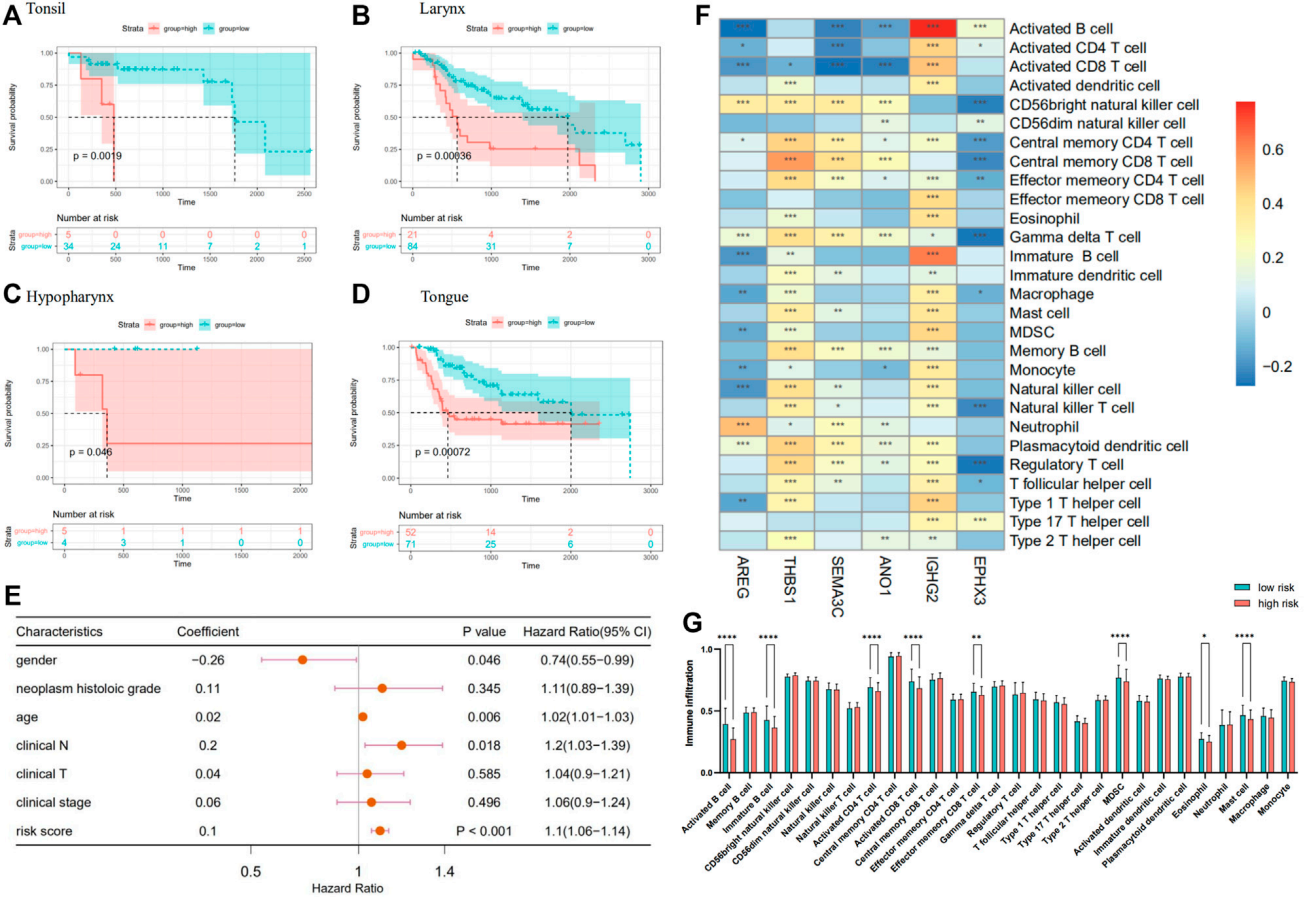
**(A–D)** A Kaplan–Meier plot establishes the survival differences between high- and low-risk groups in HNSCC with different primary sites. **(E)** Forest plot of other clinical characteristic and risk scores on a univariate Cox regression analysis. **(F)** Heatmap plotted by the correlations between the expression of genes in prognostic model and immune infiltrate level in HNSCC. **(G)** Comparison of immunocyte infiltration between high- and low-risk groups.

We performed survival analysis on 483 HNSCC patients, which reorganized according to the location of the primary tumor in an attempt to verify the reliability of the prognostic model. In several regions with high incidence of HNSCC, such as tongue and larynx (Figures 5B,D), survival comparison revealed that the high-risk group was associated with a worse prognosis of the patients. The same went for tonsil, hypopharynx, and so on (Figures 5A,C). Univariate Cox regression analyses indicate that the risk score, similar to other clinical characteristics, such as age, could be deemed as an independent risk factor to assess the prognosis of patients with HNSCC (Figure 5E). Additionally, ssGSEA was used to estimate the immune cell infiltration in the patients. To explore the correlation between prognostic model and immune cell infiltration, correlation analysis was performed between six optimal prognostic signatures and the immunocyte infiltration score (Figure 5F). Furthermore, significantly decreased infiltration of eight specific immune cells was observed in the high- compared with the low-risk group, that is, activated B cells, immature B cells, activated CD4^+^ T cells, activated CD8^+^ T cells, effector memory CD8^+^ T cells, MDSCs, and mast cells (Figure 5G).

### External Independent Cohort Validation of the Fibrosis–Hypoxia–Glycolysis–Related Prognostic Model in the GEO Data set

**FIGURE 6:**
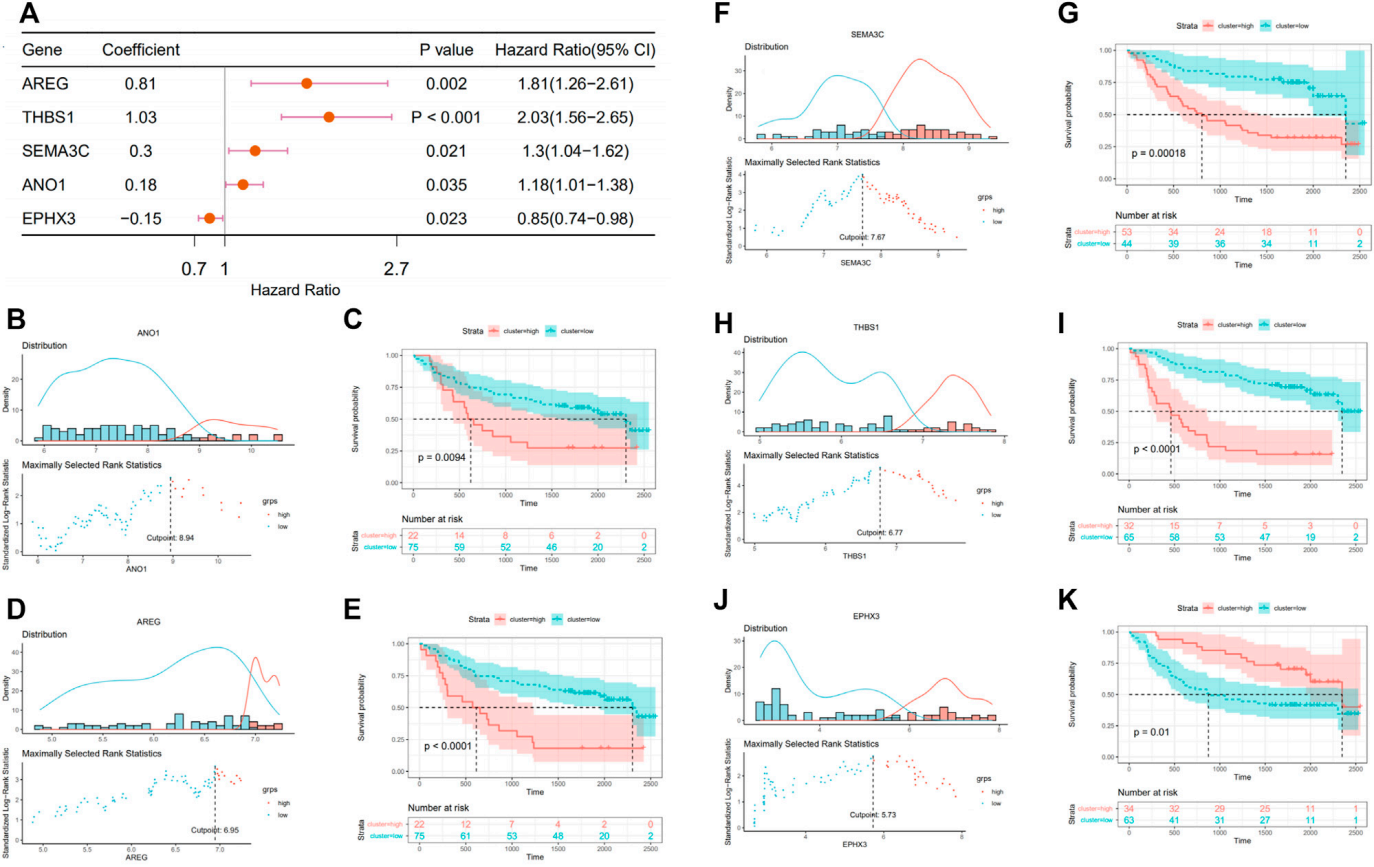
Validation in external data set (GSE416130). **(A)** Forest plot of the prognostic effects of five genes in prognostic model on a univariate Cox regression analysis. **(B,D,F,H,J)** A vertical dashed line was used to mark the cutoff point with the maximum standard log-rank statistic based on the expression of each gene. **(C,E,G,I,K)** Kaplan–Meier plot established the survival differences between high (high expression of target gene) and low (low expression of target gene) groups.

The fibrosis–hypoxia–glycolysis–based prognosis model was further validated in an independent cohort “GSE41613.” Searching for six genes from the prognosis model in the expression matrix of 97 OSCC patients, five of them were found. The clinical information of patients is shown in Table 2. Univariate Cox regression analyses confirmed that AREG, THBS1, SEMA3C, and ANO1 were risk factors in the prognosis of patients. On the contrary, EPHX3 was a protective one, which is consistent with the model previously conducted (Figure 6A). Based on maximally selected rank statistics, patients were classified into two groups according to the level of each gene expression (Figures 6B,D,F,H,J), and survival analysis was performed (Figures 6C,E,G,I,K). Kaplan–Meier curves showing that patients with higher expression levels of four risk genes in the prognostic model had worse OS (Figures 6C,E,G,I), whereas the opposite was true for protective genes (Figure 6K).

**TABLE 2:** Basic information of OSCC patients in validation cohort.

Characteristics	Whole cohort (97)
Gender	
Male	66 (0.680)
Female	31 (0.320)
Age	
≥60 years	47 (0.485)
<60 years	50 (0.515)
treatment	
uni-modality	43 (0.443)
multii-modality	53 (0.546)
unknown	1 (0.010)
tumor stage	
I/II	41 (0.423)
III/IV	56 (0.577)

### IHC Analysis of Prognostic Signatures

**FIGURE 7:**
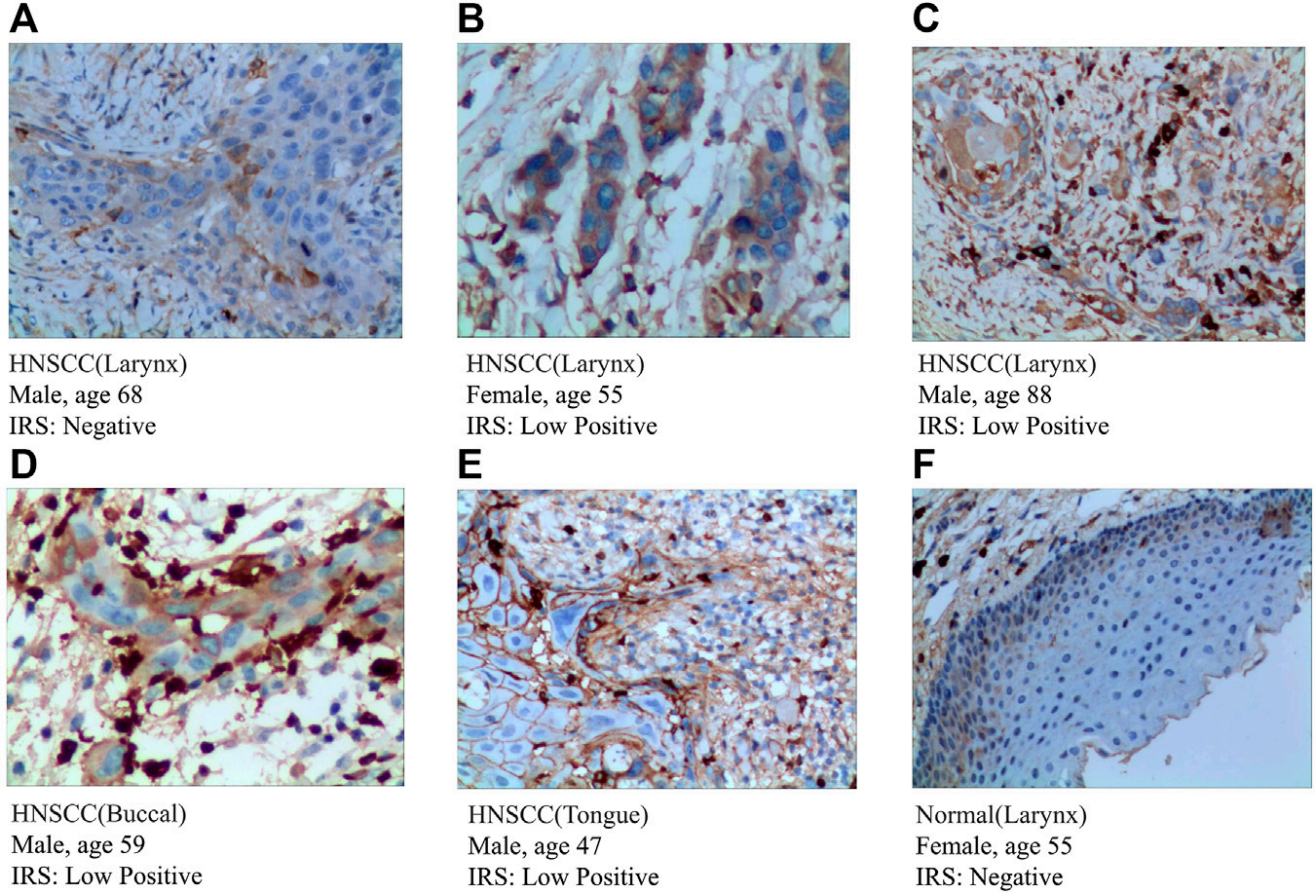
IHC analysis of the protein expression of SEMA3C in HNSCC and normal tissues. **(A–E)** The expression of SEMA3C was detected by IHC in five patients with HNSCC (Magnification ×200). **(F)** The expression of SEMA3C was detected by IHC in normal head and neck squamous cell tissue (Magnification ×200).

**FIGURE 8:**
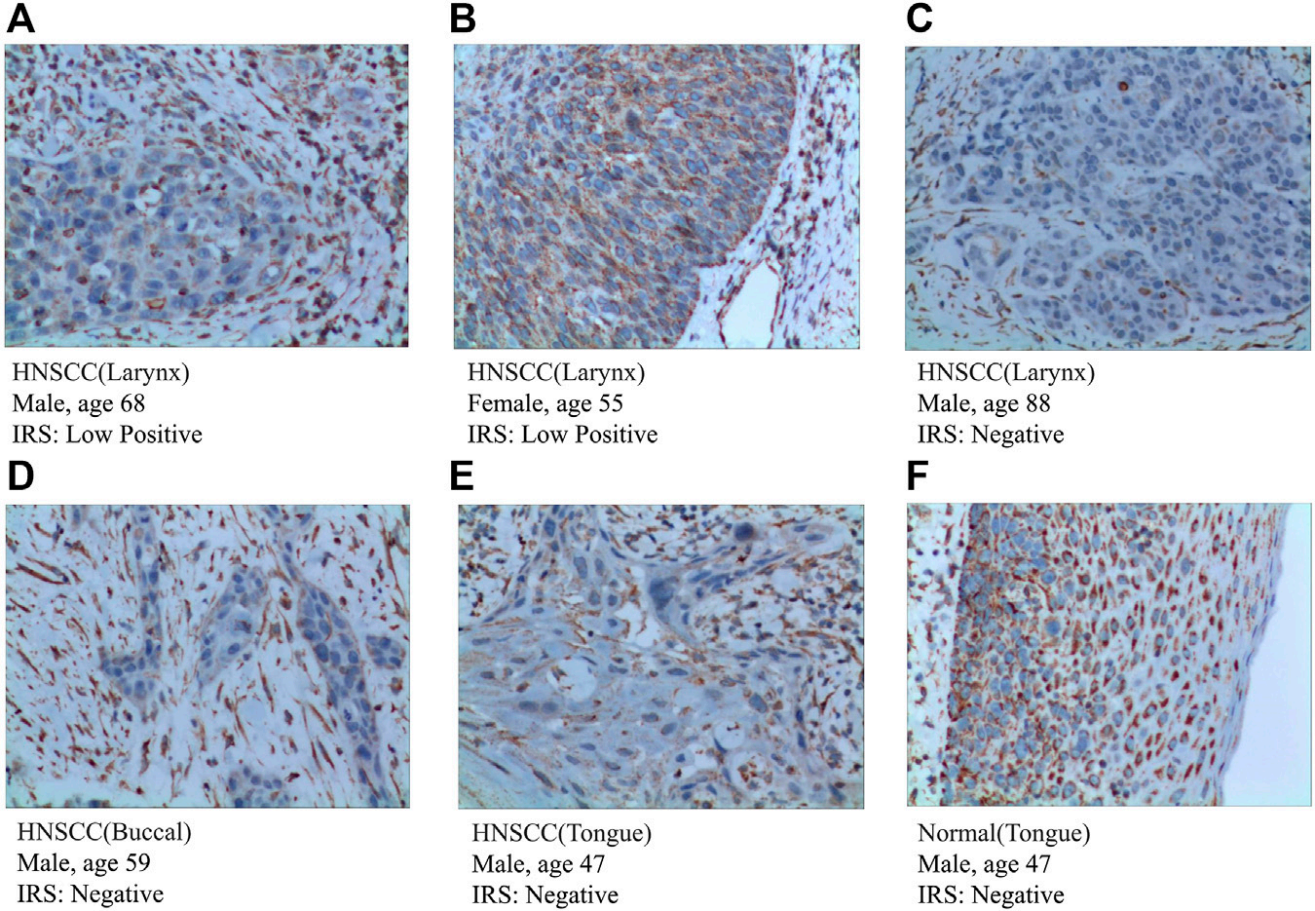
IHC analysis of the protein expression of IGHG2 in HNSCC and normal tissues. **(A–E)** The expression of IGHG2 was detected by IHC in five patients with HNSCC (Magnification ×200). **(F)** The expression of IGHG2 was detected by IHC in normal head and neck squamous cell tissue (Magnification ×200).

To further validate the expression of prognosis-related molecules in HNSCC specimens and normal squamous epithelium of head and neck, we performed IHC staining analysis of paraffin section of HNSCC. IHC staining analysis suggested that the expression of SEMA3C and IGHG2 were slightly higher in HNSCC tissue quantified by the antibodies ab135842 (Figures 7A–F) and ab134050 (Figures 8A–F).

According to the protein expression data from the HPA, we compared the protein expression of six-gene signatures in HNSCC tissue and squamous epithelium normally located in the head and neck, such as oral squamous epithelium. We preliminarily inferred that the protein expression of these genes differed between HNSCC and normal tissues. The detailed results are presented in Supplementary Figure S1.

### Fibrosis-Related Immune Landscape of HNSCC Patients Based on scRNA-Seq

**FIGURE 9:**
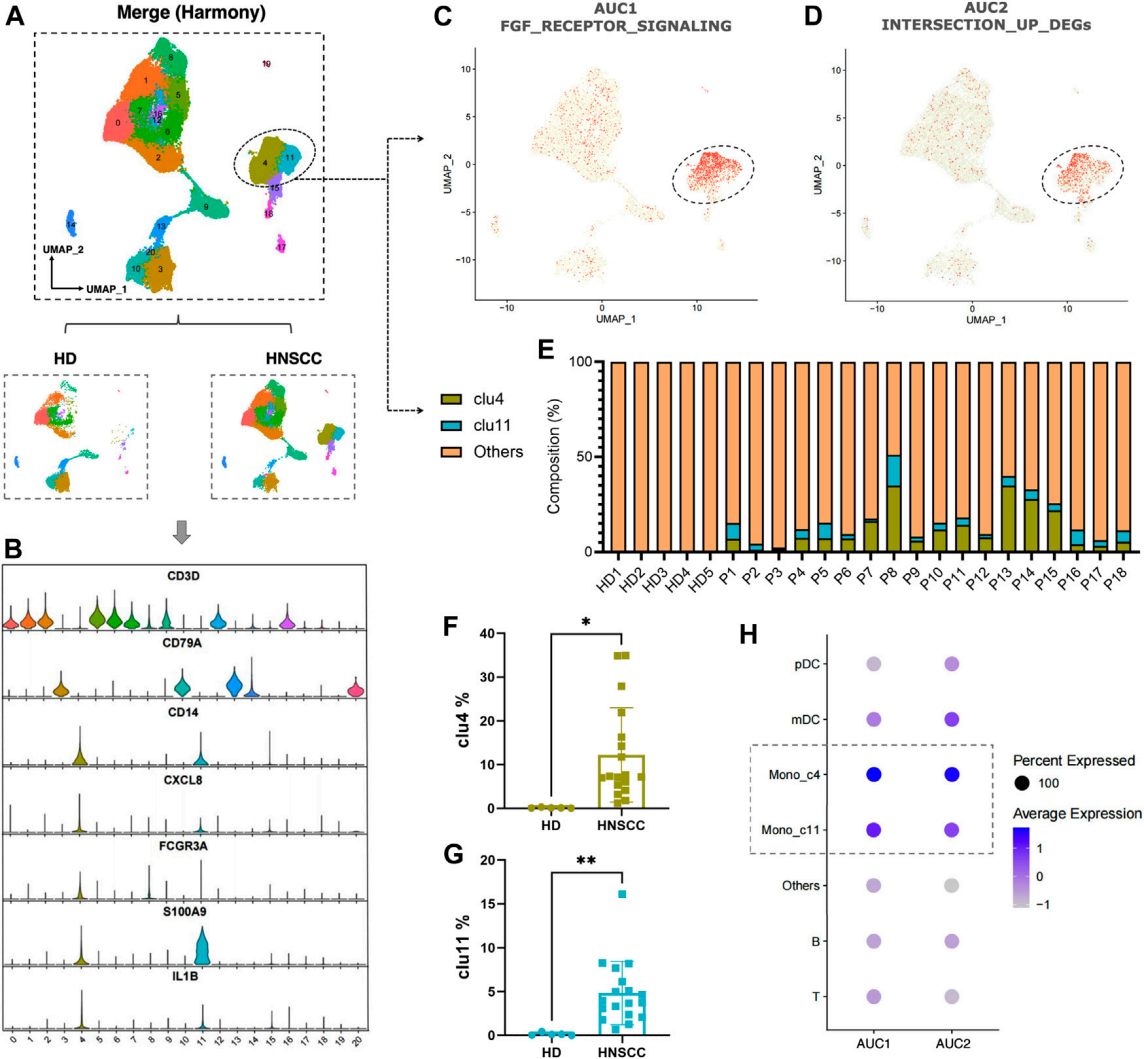
**(A)** Twenty cell clusters were successfully classified by t-SNE algorithm. **(B)** Expression of several cell surface and intracellular markers in each cell subpopulation. **(C,D)** Individual cell AUC score overlay for FGF receptor signaling and highly expressed DEGs in low-survival patients (cells from *n* = 18 HNSCC, *n* = 5 HD). **(E)** Composition of clusters 4 and 11 in each sample. **(F)** Comparison of cluster 4 between HNSCC and HD. **(G)** Comparison of cluster 11 between HNSCC and HD. **(H)** Dot plot heatmap showing AUC1 and AUC2 enriched in monocyte (clusters 4 and 11).

To further understand the correlation between the fibrosis signal and immunity, we analyzed scRNA-seq data downloaded from the GEO data set “GSE139324.” A total of 23 samples were used for analysis. Of these, 18 samples were tumor-infiltrating immune cells from HNSCC patients (HPV negative), and five were tissue-resident immune cells from healthy donor tonsils. After integrating data by the harmony algorithm and binning by the t-SNE algorithm, 20 cell clusters were successfully classified. Moreover, there was a significant difference in the number of each cell subset between HNSCC patients and healthy donors (HD) (Figure 9A). By the expression of several cell surface and intracellular markers, clusters 4 and 11 were defined as monocytes. Clusters 3, 9, 13, 14, and 20 expressed markers associated with B cells (e.g., CD79A), and the rest of the clusters expressed genes associated with T cells (e.g., CD3D) (Figure 9B). The cells with a high expression of FGF-receptor-signaling–related genes and highly expressed DEGs in low-survival patients were severally highlighted in Figures 9C,D, most of which were highly expressed in clusters 4 and 11. An obviously higher composition of monocytes is shown in patients compared with healthy controls (Figure 9E). Significant differences in the number of cells in clusters 4 and 11 were witnessed between HNSCC and HD (Figures 9F,G). The dot-plot heatmap implies that the genes mentioned are more enriched in monocytes than in other kinds of immune cells (Figure 9H).

## Discussion

Considering the poor therapeutic effect and prognosis of patients with HNSCC, it is necessary to construct an accurate prognostic staging system, which might bring personalized treatment and timely follow-up to them. In this study, a total of 483 HNSCC patients from the TCGA data set were included in our discovery cohort to exploit the prognostic value of FGFR-signal, hypoxia, and glycolysis. The effect of immune status of the tumor microenvironment (TME) was also the focus of our research. We found that profibrotic signaling, hypoxia, and glycolysis were associated with the survival of patients with HNSCC. Furthermore, we constructed a new fibrosis–hypoxia–glycolysis–related prognostic classifier including a six-gene signature for HNSCC patients under the guarantee of an external independent validation cohort. IHC was used to determine the protein expression of these genes in HNSCC tissues. The single-cell analysis of monocyte infiltration also revealed marked differences between HNSCC patients and healthy controls. These findings represent a new insight into the prognosis and tumor immune microenvironment of patients with HNSCC.

Multiple studies suggest that the FGFR signal, hypoxia, and glycolysis play a critical role in the tumorigenesis and progression of HNSCC. On the one hand, it is reported that FGFR1 amplification is a frequent event (Göke et al., 2013) and might act as a candidate prognostic biomarker in primary and metastatic HNSCC (Koole et al., 2016). Rogaratinib, an inhibitor that effectively and selectively inhibits pan-FGFR, presents a broad antitumor activity in the FGFR-overexpressing preclinical HNSCC model, revealing the potentially important role of FGFR in disease development (Grünewald et al., 2019). In the mechanism of drug resistance in head and neck cancer stem cells, the FGFR signal also shows latent vitality (McDermott et al., 2018). In addition, the FGFR signal exerts high correlation with sustaining proliferative signaling, resisting cell death, inducing angiogenesis, and activating invasion by cell migration (Ipenburg et al., 2016). However, FGFR signal–related prognostic study was still lacking in HNSCC. On the other hand, to support rapid and unlimited proliferation, solid cancer cells adopt unique energy metabolism properties, such as anaerobic glycolysis (Pavlova and Thompson, 2016) with the faster rate of ATP production and reducing the generation of reactive oxygen species (ROS) mainly produced by the electron transport chain (ETC) in the mitochondria during respiration (Yamamoto et al., 2017). This rule was also applied to HNSCC; glycolysis occurs in HNSCC cells along with a hypoxia microenvironment (Zhu et al., 2017). In the current analysis, hypoxia and glycolysis were presented as risk factors in HNSCC, which was consistent with previous studies. The FGFR signal, hypoxia, and glycolysis play a synergistic role in HNSCC prognosis. Thus, the fibrosis signal, hypoxia, and glycolysis accompanied with their interaction and its relationship with the development of HNSCC could provide improved special insight about the prognosis.

The results of single-cell analysis show that the infiltration of monocytes in the HNSCC group was higher than the HD group. Fibroblasts could communicate with the tumor cells by secreting cytokines in TME. It is reported that monocyte chemotactic protein (MCP)-1, a kind of cytokine associated with poor long-term survival of HNSCC patients (Ji et al., 2014), could be produced by fibroblasts infiltrating in TME to facilitate the recruitment of monocytes into the local inflammatory tissues and regulate their functions (Kondoh et al., 2019). This provides an explanation for the increased infiltration of monocytes in the tumor group and its close interconnection with the FGFR signal.

Significant roles of the predictive signature genes identified above are reported previously in diversified types of cancers. AREG, a ligand of epidermal growth factor receptor (EGFR), is abnormally expressed in multiple types of cancers, such as pancreatic cancer, implicated in mediating the motility, metastasis, and proliferation of tumor cells (Liu et al., 2021). It is proved that the AREG mRNA levels in cancer cells was significantly correlated with the metastatic phenotype of HNSCC tissues (Zhang et al., 2015). THBS1, known as encoding thrombospondin 1, plays a vital role in angiogenesis and tumor progression, overexpression of which was significantly associated with tumor differentiation (Yang et al., 2020). TGFB1 was reported to induce the expression of THBS1, resulting in stimulating migration of cancer cells and driving the expression of MMP3 (matrix metalloprotease 3) *via* integrin signaling, conducive to OSCC intrusion (Pal et al., 2016). Though the activation of the p-ERK pathway, SEMA3C promotes cervical cancer growth, which is related to poor prognosis (Liu et al., 2019a). ANO1 encodes a calcium-dependent chloride channel protein and commonly amplifies to facilitate several cancers’ progression, including ovarian (Liu et al., 2019b), prostate (Liu et al., 2012), breast (Britschgi et al., 2013), and head and neck cancers (Filippou et al., 2021). After neoadjuvant chemoradiotherapy, the expression level of IGHG2 increased significantly in rectal cancer, indicating that IGHG2 was originally with a low expression in tumor cells and existed as a protective factor, which was consistent with our prognostic analysis. The protective gene EPHX3, known as epoxide hydrolase 3, whose hypermethylation is responsible for the development of OSCC (Morandi et al., 2017), contributes to predict the survival of HNSCC patients (Bai et al., 2019). However, six signature genes in this study were barely mentioned in the context of combination of the FGFR signal, hypoxia, and glycolysis. Thus, the abovementioned signature genes could provide therapeutic targets and directions for the elucidation of molecular mechanisms in HNSCC.

There were inevitable limitations in this study. The first limitation is that IGHG2 was not detected in the independent external cohort, so we could not validate the effect of it. More independent HNSCC cohorts should be used for the validation of the established prognostic model. Using expression profiles downloaded from publicly available databases, it is difficult for us to ensure that the validation samples including all primary tissue sites of tumors in TCGA. Thus, verification of findings above requires more well-designed, comprehensive, and thorough study.

## Conclusion

In conclusion, the status of the FGFR signal, hypoxia, and glycolysis correlate with the prognosis of HNSCC patients. The prognostic model conducted above might provide potential application value for prognosis prediction and individualized treatment.

## Data Availability

The data sets presented in this study can be found in online repositories. The names of the repository/repositories and accession number(s) can be found in the article/Supplementary Material.
