# Supplementary figures and images for: Investigation of an FGFR-Signaling-Related Prognostic Model and Immune Landscape in Head and Neck Squamous Cell Carcinoma
Source: Front Cell Dev Biol. 2022 Feb 14;9:801715. doi: 10.3389/fcell.2021.801715 (PMC8882630; doi:10.3389/fcell.2021.801715)

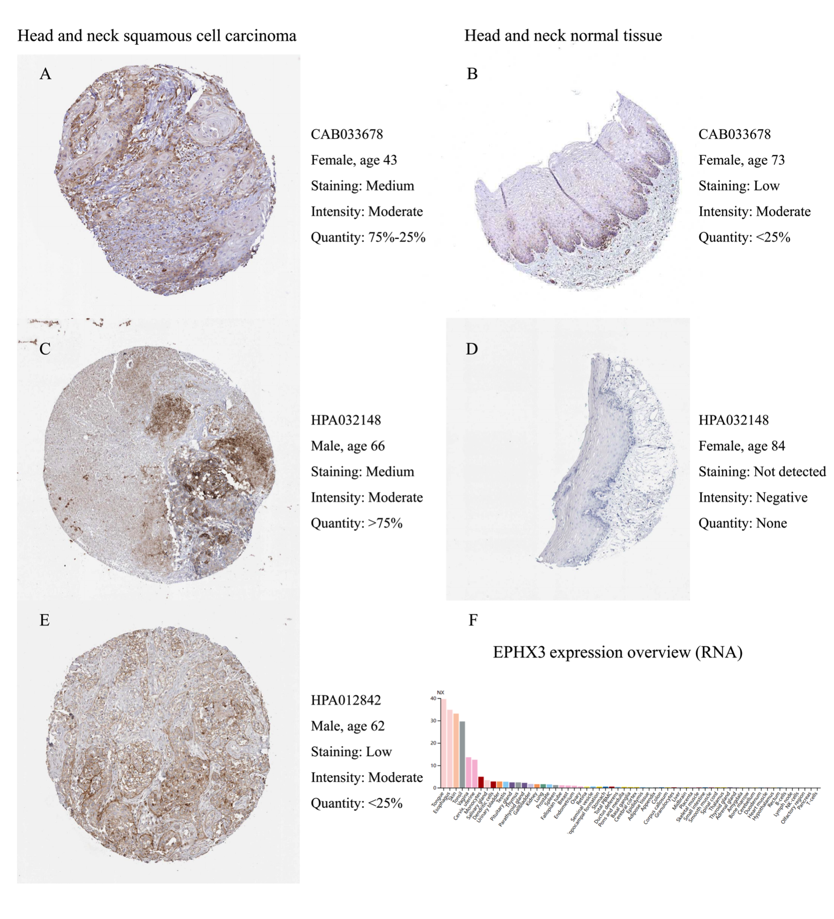

Supplement: Supplementary file 1 [file Image1.tiff]
